# *Solanum aethiopicum* L. from the Basilicata region as a source of specialized metabolites with promising anti-obesity effects: phytochemical characterization and *in vivo* investigation in high fat diet-fed mice

**DOI:** 10.3389/fphar.2023.1306135

**Published:** 2023-11-22

**Authors:** Maria Ponticelli, Laura Hidalgo-García, Patricia Diez-Echave, Teresa Vezza, Miguel Romero, Iñaki Robles-Vera, Juan Duarte, Filomena De Biasio, Domenico Gorgoglione, Ludovica Lela, Julio Galvez, Luigi Milella

**Affiliations:** ^1^ Department of Science, University of Basilicata, Potenza, Italy; ^2^ Department of Pharmacology, Center for Biomedical Research (CIBM), University of Granada, Granada, Spain; ^3^ Instituto de Investigación Biosanitaria de Granada (ibs.GRANADA), Granada, Spain; ^4^ Servicio de Digestivo, Hospital Universitario Virgen de Las Nieves, Granada, Spain; ^5^ Centro de Investigación Biomédica en Red de Enfermedades Cardiovasculares (CIBERCV), Instituto Salud Carlos III, Madrid, Spain; ^6^ Evra S.r.l. Benefit Company, Lauria, Italy; ^7^ Centro de Investigación Biomédica en Red de Enfermedades Hepáticas y Digestivas (CIBEREHD), Instituto Salud Carlos III, Madrid, Spain

**Keywords:** inflammation, obesity, scarlet lucanian eggplant, *Solanum aethiopicum* linn, type 2 diabetes

## Abstract

**Introduction:** *Solanum aethiopicum* L., commonly known as scarlet eggplant (Solanaceae family) is one of the most traditionally cultivated vegetables in Basilicata, a southern region of Italy. Although multiple uses have been given to this vegetable, data about its anti-obesogenic activity are still limited.

**Methods:** This study focuses on testing two different extracts obtained either from the peel or from the whole fruit of the Lucanian *Solanum aethiopicum.* Their ability to inhibit certain enzymatic activities was tested *in vitro* and then, the one that showed the better outcomes was tested on an experimental model of High-Fat Diet (HFD) induced obesity.

**Results:** Spectrophotometric assays demonstrated that the peel extract possessed the highest ability to inhibit the selected enzymatic activities and so, its phytochemical profile was obtained through LC-MS chromatography. The oral administration of this extract (25 mg/kg) to HFD-fed mice reduced body weight gain and improved glucose and lipid metabolism. Similarly, the extract ameliorated the obesity-induced inflammatory status by reducing the expression of pro-inflammatory cytokines in both adipose and hepatic tissues. Interestingly, these effects were associated with the improvement of vascular dysfunction.

**Discussion:** Lucanian *Solanum aethiopicum* extract may represent a new strategic approach for managing obesity and its associated diseases.

## 1 Introduction

Overweight and obesity are complex, multifactorial, and largely preventable diseases considered to be a global epidemic as they affect over a third of the world’s current population. The increase in weight gain is caused by an imbalance between energy expenditure and energy intake (Van Dam and Seidell, 2007), leading to excessive growth and expansion of adipose tissue, associated with an impaired release of adipokines and pro-inflammatory cytokines (Sinisgalli et al., 2021); in fact, obesity is considered as a chronic low-grade inflammatory disease (Cancello et al., 2005). Moreover, it significantly impacts health as it is related to the onset of serious complications such as type 2 diabetes, cardiovascular diseases, and cancer, causing enhanced morbidity and premature mortality (Li et al., 2022). Nowadays, obesity management is associated with exercise and dietary modifications, and in some cases, with pharmacotherapy or even surgery. However, drastic dietary interventions and exercise are hardly accepted by obese patients, while bariatric surgery exposes patients to serious risks. Therefore, pharmacotherapy usually represents the selected strategy for helping obese patients to lose weight; nevertheless, the lack of efficacy of some of these drugs, their high cost and/or the incidence of adverse effects can limit their use (Saunders et al., 2018). For this reason, the use of alternative and/or complementary therapies has been long considered in obesity treatment, including those from natural products that could be safe and effective (Bahmani et al., 2016). In fact, several studies have reported the potential antiobesity effect of plant extracts, mainly carried out in experimental models in rodents (Vijayalatha, 2004; Diez-Echave et al., 2020; Diez-Echave et al., 2020; Sinisgalli et al., 2021; Molina-Tijeras et al., 2023).

The *Solanum aethiopicum* L. or scarlet eggplant is a vegetal species belonging to the Solanaceae family and is one of the main vegetables consumed in tropical Africa. Together with onion, tomato, okra, and pepper, it is among the five most predominantly cultivated crops in Central and West Africa, being also grown in the Caribbean and Brazil (Han et al., 2021)*.* In Italy, *Solanum aethiopicum* populations have been cultivated only in Basilicata, specifically in the Regional Natural Reserve of Pollino, where it has been consumed for several years together with the brinjal eggplant (*Solanum melongena*) (Sunseri et al., 2010). To date, this Italian aubergine has participated in the Slow Food Foundation catalogue and joined the Protected Designation of Origin category (DOP) (De Cristofaro et al., 2014).

Many studies have reported the biological activities of the African eggplant, including antidiabetic, anti-inflammatory, hepatoprotective, and nephroprotective effects (Nwanna et al., 2019a). In particular, eggplant species have been largely used for managing diabetes in traditional medicine, and the Mayo Clinic and American Diabetes Association have also advised using this eggplant as a vegetable for the management of degenerative conditions (Association, 2014; Nwanna et al., 2016). These beneficial properties are possibly attributed to its phytochemical composition since *S. aethiopicum* has been revealed to be a source of phenolic compounds, carotenoids and alkaloids (Sanchez-Mata et al., 2010; Gürbüz et al., 2018; Mbondo et al., 2018) with known anti-inflammatory, antioxidant, antiatherosclerotic, and anti-obesogenic properties (Gürbüz et al., 2018; Han et al., 2021). Considering this promising background, and the need for additional information about the beneficial effects exerted by African eggplant, the present study aimed to test the activity of the Lucanian *S. aethiopicum* peel and whole fruit ethanolic extracts in inhibiting the activity of enzymes involved in the regulation of weight gain. Furthermore, the extract with the best activity was phytochemically characterized by LC-MS and assayed *in-vivo* in an experimental model of obesity in mice induced by a HFD intake.

## 2 Materials and methods

### 2.1 Chemicals and reagents

LC-ESI/LTQOrbitrap/MS solvents were purchased from VWR (Milan, Italy), while formic acid and acetonitrile were purchased from Merck (Merck KGaF, Darmstadt, Germany). *α-*glucosidase (maltase from *Saccharomyces cerevisiae*), *α-*amylase (from porcine pancreas), acarbose, quercetin, 4-Nitrophenyl *α*-D-Glucopyranoside (PNP-G) and soluble starch, β-mercaptoethanol, Tris-HCl, trisodium phosphate (Na_3_PO_4_), potassium sodium tartrate tetrahydrate (KNaC_4_H_4_O_6._4H_2_O), sodium hydroxide (NaOH), DL-glyceraldehyde and *tert*-Butyl Methyl Ether (MTBE) were from Sigma-Aldrich (st. Louis, MO, United States of America). All chemicals used for the *in vivo* studies were purchased from Sigma-Aldrich Quimica SL (Madrid, Spain).

### 2.2 Sample preparation

The investigated *S. aethiopicum* species were grown in Basilicata Region (Italy) and provided by Evra S.r.l. Benefit Company (Località Galdo, Lauria PZ). Whole fruit and peel were reduced to small pieces and extracted using the technique of exhaustive maceration. The plant material (234 g in the case of the peel and 124 g in the case of the entire fruit) was extracted with absolute ethanol (plant material:solvent ratio 1:20) and stored in the dark for 48 h at room temperature. The extraction procedure was made three times. The obtained extracts were filtered, and the solvent was evaporated through a rotary evaporator. The obtained dried extracts were protected from light at ambient temperature until their use.

### 2.3 α-amylase and α-glucosidase assays

The ability of *S. aethiopicum* extracts to inhibit *α*-glucosidase and *α*-amylase activities was determined spectrophotometrically in Multiskan GO (Thermo Scientific, Finland) equipped with the Skanlt 4.1 software. Both assays were performed following the method previously described by Figueiredo-González et al. (2016). Acarbose, an *α-*amylase and *α*-glucosidase inhibitor, was used as a positive control and experimental assays were performed in triplicate.

### 2.4 Aldose Reductase assay

Aldose Reductase (AR) activity was assessed by measuring the decay of NADPH absorption at 340 nm, in accordance with the method described by Suryanarayana et al. (2004), with slight modifications. Briefly, equal volumes (40 μL) of AR (0.052 U/mL), DL-glyceraldehyde (10 mM), and extract solution were pre-incubated at 37°C for 2 min on a 96-well plate. The reaction was initiated by adding 80 μL of NADPH (0.5 mM), and the change of absorbance was followed for 20 min. Samples and reagents were prepared in 0.1 M phosphate buffer with 5 mM β-mercaptoethanol and 0.2 mM ammonium sulphate (pH 6.2). AR inhibition was determined by measuring the decrease in absorbance caused by the extract when compared with that found in the control (only buffer). The assay was performed in triplicate. Rutin, a flavonoid glycoside, was used as a positive control.

### 2.5 LC-ESI/LTQOrbitrap/MS

The phytochemical characterization of *S. aethiopicum* peel extract was performed using an HPLC coupled with a mass spectrometer following the method of Sinisgalli *et al.* (2020).

### 2.6 *In vivo* assays

#### 2.6.1 Experimental animals and diets

The study was carried out following the “Guide for the Care and Use of Laboratory Animals” as assessed by the National Institute of Health and the protocols endorsed by the Ethic Committee of Laboratory Animals of the University of Granada (Spain) (Ref. No. 28/03/2016/030). 5-week-old C57BL/6J male mice (Janvier, St Berthevin, Cedex, France) were housed in standard conditions (12-h light/dark cycle, temperature 22°C ± 1°C, 55% ± 10% relative humidity) with free access to food and water. Mice were randomly assigned to one of the following groups: lean control (SD) (n = 8), obese (HFD) (n = 8), obese treated with *S. aethiopicum* (HFD-*S. aethiopicum*) (n = 8) and obese treated with metformin (HFD-Metformin) (n = 8). Control mice received a standard chow diet (SD group) (13% calories from fat, 20% calories from protein, and 67% calories from carbohydrate; Global diet 2014; Harlan Laboratories, Barcelona, Spain), whereas obese mice were fed a high-fat diet (HFD group) in which 60% of its caloric content was derived from fat (47% monosaturated, 37% saturated, 16% polyunsaturated; Purified diet 230 HF, Scientific Animal Food and Engineering, Augy, France).

#### 2.6.2 Dosage information

The obese-treated mice (HFD-*S. aethiopicum* group) were daily administered by oral gavage with the ethanolic extract of the Lucanian *Solanum aethiopicum* peel dissolved in water (25 mg/kg), for a total period of 5 weeks. The lean mice (SD-group) only received the vehicle, instead. Additionally, metformin (250 mg/kg/day) treated mice were used as a positive control group in macroscopic and plasma biochemical determinations. Animal body weight, water, and food intake were controlled regularly during the whole experiment.

#### 2.6.3 Glucose tolerance test, plasma determinations and morphological variables

A glucose tolerance test was carried out 1 week before the end of the experiment (see a detailed protocol in Supporting Information online). Before sacrifice, mice were fasted overnight and, under isoflurane anaesthesia, blood samples were collected to determine glucose, Low-Density Lipoprotein (LDL)-cholesterol, High-Density Lipoprotein (HDL)-cholesterol, and total cholesterol. Liver, adipose, and colonic tissue were extracted to evaluate gene expression by RT-q-PCR (Supplementary Table S1, Supporting Information).

#### 2.6.4 Vascular reactivity studies and NADPH oxidase activity

The obesity-associated vascular dysfunction was performed in descending thoracic aortic rings by measuring acetylcholine vaso-relaxant ability and NADPH oxidase activity (see a detailed protocol in Supporting Information online).

### 2.7 Statistical analysis

*In vitro* experiments were performed in triplicate and the results were reported as mean ± SD. *In vivo* studies were expressed as mean ± SEM. Differences between mean were analysed for statistical analysis by using *post hoc* least significance tests and one-way analysis of variance (ANOVA). The software used was GraphPad 9.0 software package (GraphPad Software, Inc., La Jolla, CA, United States of America); statistical significance was set at *p < 0.05*.

## 3 Results and discussion

### 3.1 Enzymatic assays

**FIGURE 1:**
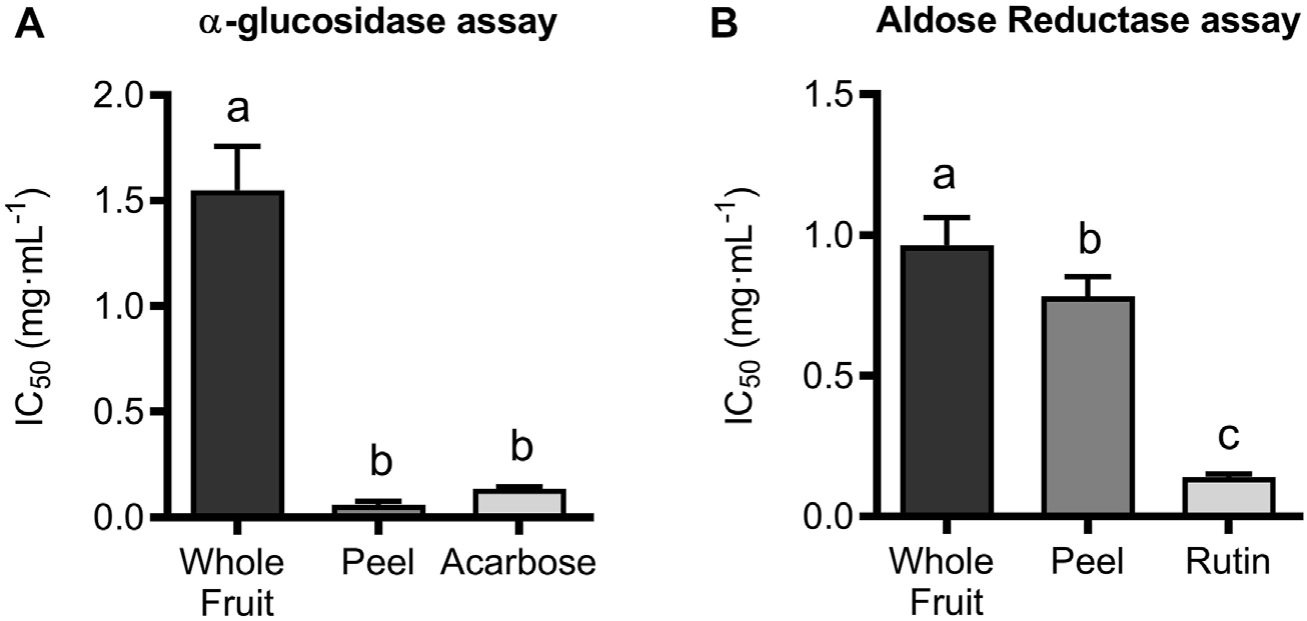
Enzymatic inhibition expressed as IC_50_ of ethanolic (EtOH) extract of *S. aethiopicum* whole fruit and peel extracts and positive control (acarbose) towards **(A)** α-glucosidase and **(B)** Aldose Reductase. Results show mean ± standard deviation (mg dry extract/mL) of at least three experiments performed in triplicate. Groups with different letters statistically differ (*p < 0.05*).

A plausible strategy to promote weight loss and prevent obesity can be related to the inhibition of the metabolism and/or absorption of carbohydrates, thereby decreasing caloric intake. It is known that before absorption, carbohydrates are broken into monosaccharides by two major enzymes, amylase, secreted by the pancreas into the small intestine, and glucosidase, located in the brush border of epithelial cells in the small intestine. The *α*-amylase hydrolyses *α*-1,4-glycosidic bonds in polysaccharides (e.g., starch, amylopectin, amylose, etc.) to form low molecular weight molecules as disaccharides and trisaccharides (e.g., maltotetraose, maltose, and maltotriose). Subsequently, *α*-glucosidase converted these maltodextrins into absorbable monosaccharide units (Mahmood, 2016; Nwanna et al., 2019a). The inhibition of *α*-amylase or *α*-glucosidase activities is one of the approaches for managing hyperglycemia in diabetic patients. In fact, it is well known that inhibitors of these enzyme activities, like acarbose, voglibose, and miglitol, reduce blood glucose levels after a meal by inhibiting starch breakdown and then monosaccharide assimilation. However, it has been reported that excessive *α*-amylase inhibition can be responsible for the colonic fermentation of undigested carbohydrates by bacteria, thus leading to the onset of classic side effects related to the administration of these drugs, including abdominal distension, flatulence, and bloating. For this reason, it has been proposed that a mild reduction of *α*-amylase activity and the strongest *α*-glucosidase inhibition should avoid these side effects while maintaining therapeutic activity (Nwanna et al., 2014). When the Lucanian extract of *S. aethiopicum* was assayed against these enzyme activities, it showed almost complete inhibition of *α*-glucosidase. In contrast, mild activity was observed for *α*-amylase (data not shown). In addition, the peel extract showed a higher potency as an inhibitor of *α*-glucosidase than the whole fruit (IC_50_ values of 0.0535 ± 0.0118 mg/mL and 1.532 ± 0.292 mg/mL, respectively). Acarbose, an intestinal α-glucosidase and pancreatic α-amylase inhibitor generally administered to treat type 2 diabetes, was used as a positive control and showed its known ability to inhibit α-glucosidase with an IC_50_ value of 0.116 ± 0.0035 mg/mL (Figure 1A). The present results confirm previous studies performed on African eggplants (Nwanna et al., 2014; Nwanna et al., 2019a), although the inhibitory activity of either Lucanian *S. aethiopicum* peel or whole fruit extract is higher than that of two different Nigerian *S. aethiopicum* fruit species (IC_50_ of 1.10 ± 0.10 mg/mL and 0.90 ± 0.02 mg/mL for Ibadan and Uyo species, respectively) (Nwanna et al., 2019a). Similarly, the Italian *S. aethiopicum* was more active than other garden eggplants commonly consumed in Nigeria, like *Solanum torvum* Swartz, *Solanum gilo* L., *Solanum incanum* L., *Solanum kumba* L., and *Solanum indicum* L., which showed IC_50_ values against α-glucosidase ranging from 1.02 to 1.04 mg/mL (Nwanna et al., 2014). In addition to *α*-amylase and *α*-glucosidase activities, it is well known that the polyol pathway plays an important pathogenic role in diabetes since it is responsible for glucose reduction in sorbitol by the AR enzyme, using NADPH as a cofactor, and thus promoting sorbitol accumulation in the cell, which causes cellular and organ damage, together with NADPH depletion and decrease of nitric oxide (NO) levels and glutathione production. Reduced NO levels may result in circulatory abnormalities, while glutathione depletion can lead to an increased accumulation of reactive oxygen species, thus worsening endothelial functions (Schemmel et al., 2010). There is a considerable effort to develop drugs targeting AR to reduce sorbitol accumulation in cells; this is the case of Tolrestat, a well-tolerated AR inhibitor used for the treatment of diabetic complications such as diabetic peripheral neuropathy. The present study showed that both, whole fruit and peel extracts from Lucanian *S. aethiopicum*, were able to inhibit AR activity, being their IC_50_ values 0.974 ± 0.128 mg/mL and 0.789 ± 0.05 mg/mL, respectively. That indicates that, like in the case of the α-glucosidase inhibition, the peel extract was more active than the whole fruit one. However, the peel extract had lower activity than the positive control, rutin (IC_50_ of 0.132 ± 0.176 mg/mL) (Figure 1B).

These results suggested that *S. aethiopicum* peel extract should be used for treating diabetes complications by targeting in particular *α*-glucosidase and AR; hence its phytochemical profile was investigated using LC-ESI-Orbitrap-MS/MS chromatography.

### 3.2 LC-MS quantification

**FIGURE 2:**
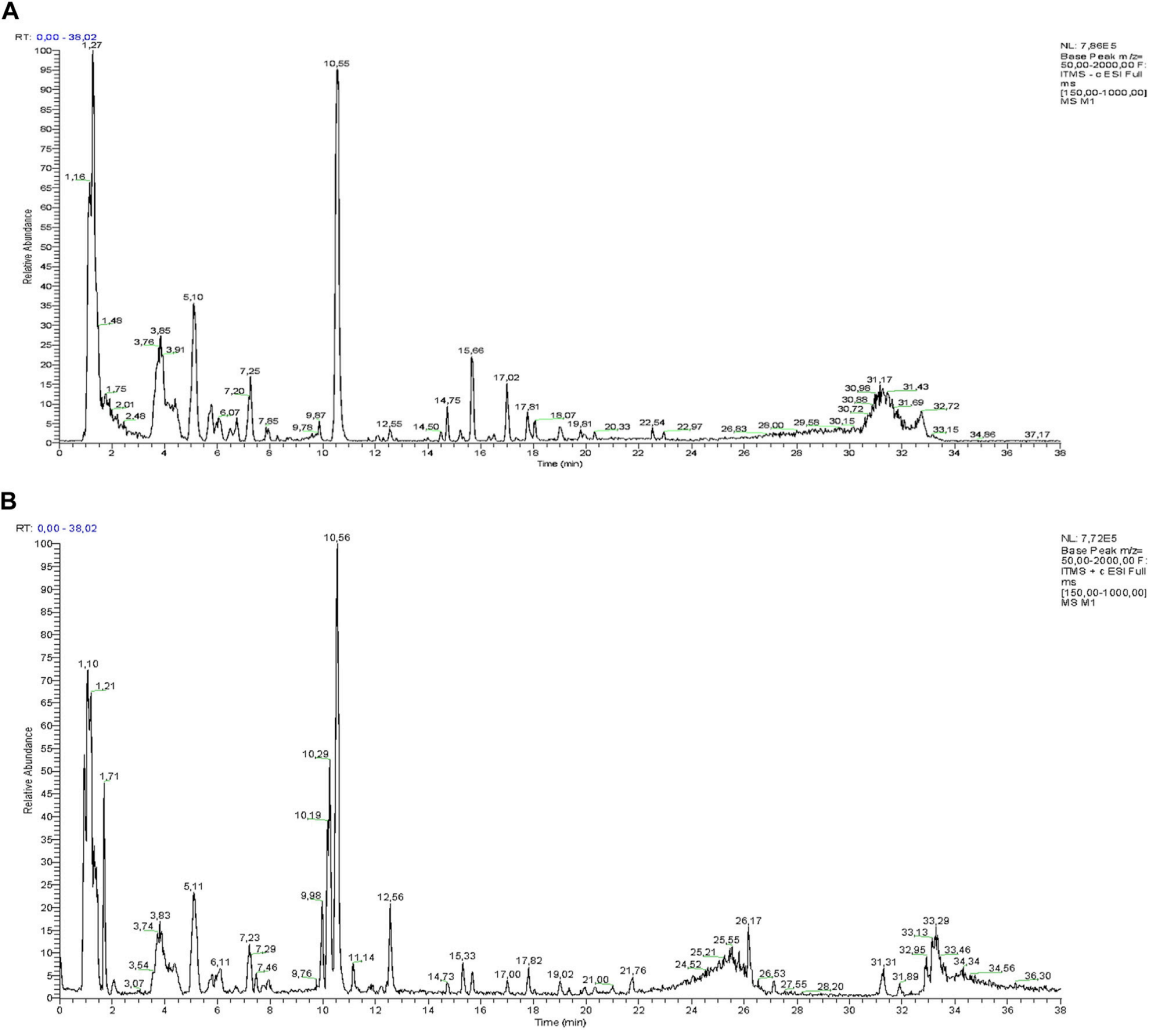
LC-MS profile of Lucanian *S. aethiopicum* L. peel ethanol extract in the negative **(A)** and positive **(B)** ion mode.

LC-ESI-Orbitrap-MS/MS provided a sensitive and fast analytical tool for the phytochemical profile analysis of the Lucanian *S. aethiopicum*. Specifically, using the Luna C18 column and LC-ESI-MS/MS combination, it was possible to separate and identify polar and non-polar compounds simultaneously by alternating positive and negative ionization modes (Figure 2).

Individual active molecules were identified by comparing their m/z values in the total ion current (TIC) profile with those of the compounds described in the literature (Nwanna et al., 2014; Mibei et al., 2017; Nwanna et al., 2019a). Thirteen compounds were identified in the Lucanian *S. aethiopicum* peel extract belonging to a wide variety of structurally different metabolic classes: phenolic acids (caffeic acid, gallic acid, ellagic acid, chlorogenic acid), flavonols (quercetin, rutin, kaempferol), flavanols (epicatechin), anthocyanins (delphinidin-3-rutinoside), vitamins (ascorbic acid), carotenoids (*β*-carotene, lycopene), and sesquiterpenoids (solavetivone) (Table 1).

**TABLE 1:** Active compounds identified in the Lucanian *Solanum aethiopicum* L. peel ethanolic extract using LC-ESI/Orbitrap/MS/MS.

Compounds	Rt (min)	Molecular formula	MW	[M + H]^+^	[M-H]^-^	Product ions (*m/z*)
*Caffeic Acid*	1.25	C_9_H_8_O_4_	180.0422		179.0344	174, 135
*Kaempferol*	1.91	C_15_H_10_O_6_	286.0477		285.0399	267, 191, 165
*Delphinidin-3-rutinoside*	3.82	C_27_H_31_O_16_	611.1612		610.1533	609, 300, 125
*Rutin*	5.85	C_27_H_30_O_16_	610.1533		609.1455	300, 179, 151
*Ellagic acid*	8.48	C_14_H_6_O_8_	302.1991		301.1970	257, 150
*Quercetin*	10.72	C_15_H_10_O_7_	302.0426		301.0348	287, 151, 121
*Epicatechin*	33.12	C_15_H_14_O_6_	290.0790		289.0712	245, 227, 165, 161
*Ascorbic acid*	33.31	C_6_H_8_O_6_	176.0320		175.0242	157, 115, 112, 87
*Gallic Acid*	1.41	C_7_H_6_O_5_	170.0215	171.0287		157, 110, 82
*Chlorogenic acid*	17.03	C_16_H_18_O_9_	354.0950	355.1029		338, 273, 227, 197, 173, 163, 147
*β-carotene*	18.18	C_40_H_56_	536.4382	537.4460		457, 444, 429, 391, 338, 273, 199, 171
*Lycopene*	18.20	C_40_H_56_	536.4382	537.4460		444, 375, 344, 309, 273, 207, 199, 171
*Solavetivone*	25.42	C_15_ H_22_O	218.1670	219.1748		207, 199, 198, 182, 171, 166, 163

The identified compounds showed the typical ion peaks reported by several studies; for example, the ellagic acid demonstrated [M-H]−ion peak at 𝑚/𝑧 301.1970 and fragment ions at 𝑚/𝑧 257 and 150 (Jiang et al., 2017). Moreover, β-carotene and lycopene exhibited molecular ions in positive ionisation mode with characteristic fragmentation patterns (444/429 for *β*-carotene and 444/375 for lycopene) (Zhang et al., 2019). Among the compounds identified, there is also the sesquiterpenoid solavetivone, a phytoalexin characteristic of the Solanaceae family (Sabater-Jara et al., 2010).

The compounds identified could corroborate the inhibitory activity evaluated on α-glucosidase since previous investigations have demonstrated that phenolic compounds like caffeic acid or quercetin showed a strong inhibitory effect on this enzyme. Specifically, it was seen that they had a high α-glucosidase active site binding interaction thanks to the ability to form links with the Arg^411^ and Arg^407^ residues directly (Rasouli et al., 2017). On the other hand, flavonols like kaempferol have demonstrated outstanding potential in interacting with the active site of *α*-amylase (Schemmel et al., 2010). Furthermore, chlorogenic acid, the main phenolic acid found in African *S. aethiopicum* (Plazas et al., 2014; Nwanna et al., 2019a), and also identified in the peel of Lucanian species, demonstrated antidiabetic activity in diabetic animal models and a strong AR inhibitory effect (Alim et al., 2017).

Considering these assumptions, the peel extract was chosen for *in vivo* studies on obese mice.

### 3.3 Experimental model of high-fat diet (HFD)-induced obesity in mice

#### 3.3.1 The administration of *S. aethiopicum* L. Peel extract to HFD-fed mice decreased weight gain and improved glucose and lipid metabolism

**FIGURE 3:**
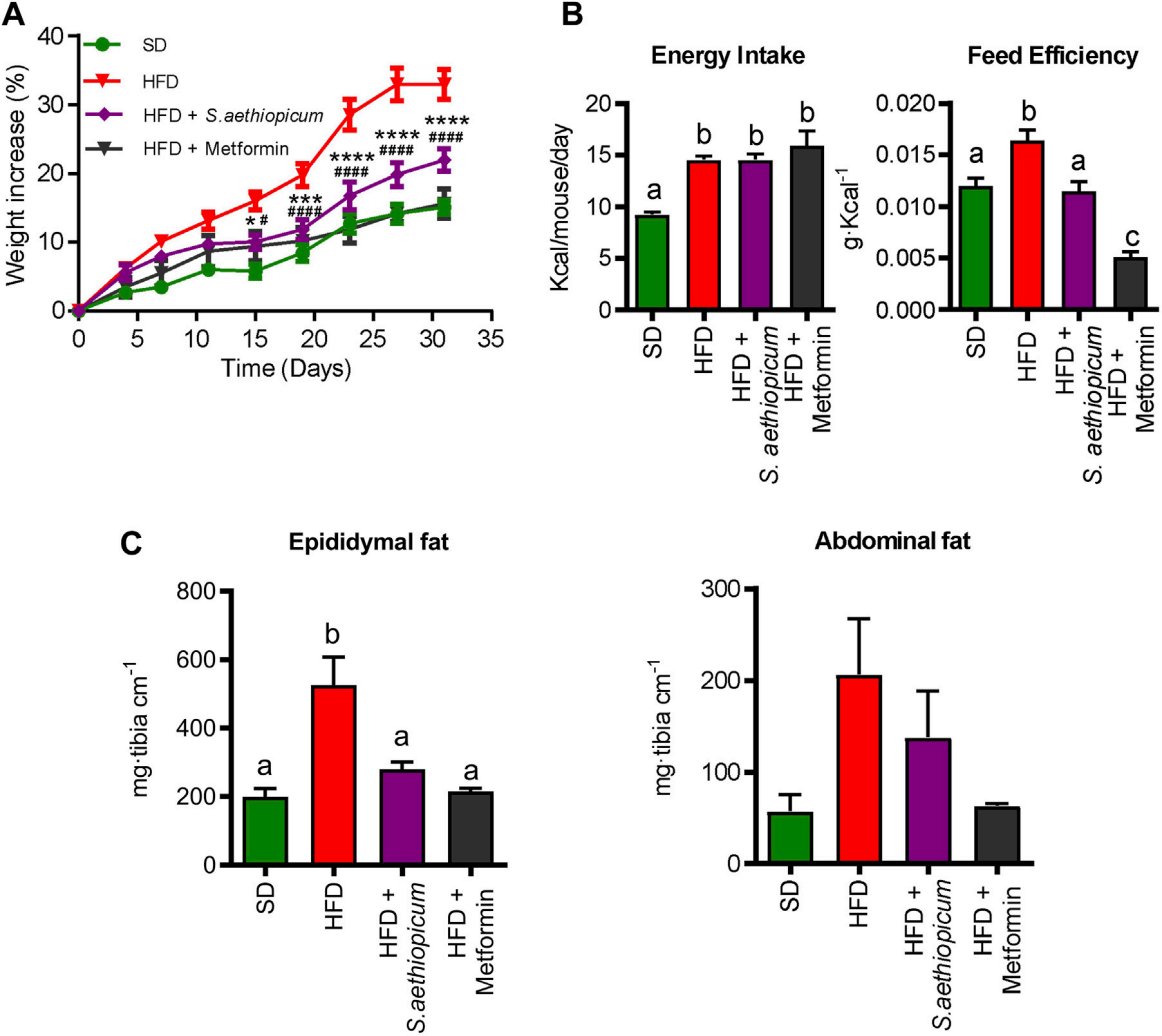
Effects of *S. aethiopicum* peel extract and metformin administration on **(A)** body weight evolution **(B)** energy efficiency and energy intake **(C)** epididymal and abdominal fat deposits weights in standard (SD) and High-Fat Diet (HFD)-fed mice. Data are expressed as means ± SEM (n = 8). Groups with different letters statistically differ (*p < 0.05*); ***p < 0.001 and ****p < 0.0001 vs. HFD-fed mice.

The Lucanian *S. aethiopicum* peel extract was tested in an experimental model of obese mice fed with a high-fat diet (HFD). As expected, the body weight gain of untreated HFD-mice was significantly higher when compared with that of non-obese mice (Figure 3A). However, the administration of *S. aethiopicum* peel extract or metformin to HFD-mice significantly reduced this weight gain without modifying total energy intake, thus lowering energy efficiency (Figure 3B). This effect was associated with a significant decrease in the adipose fat mass, since the increased weight of abdominal and epididymal adipose tissues observed in the control obese group was significantly reduced in those obese mice treated with the Lucanian eggplant extract or metformin (Figure 3C).

**FIGURE 4:**
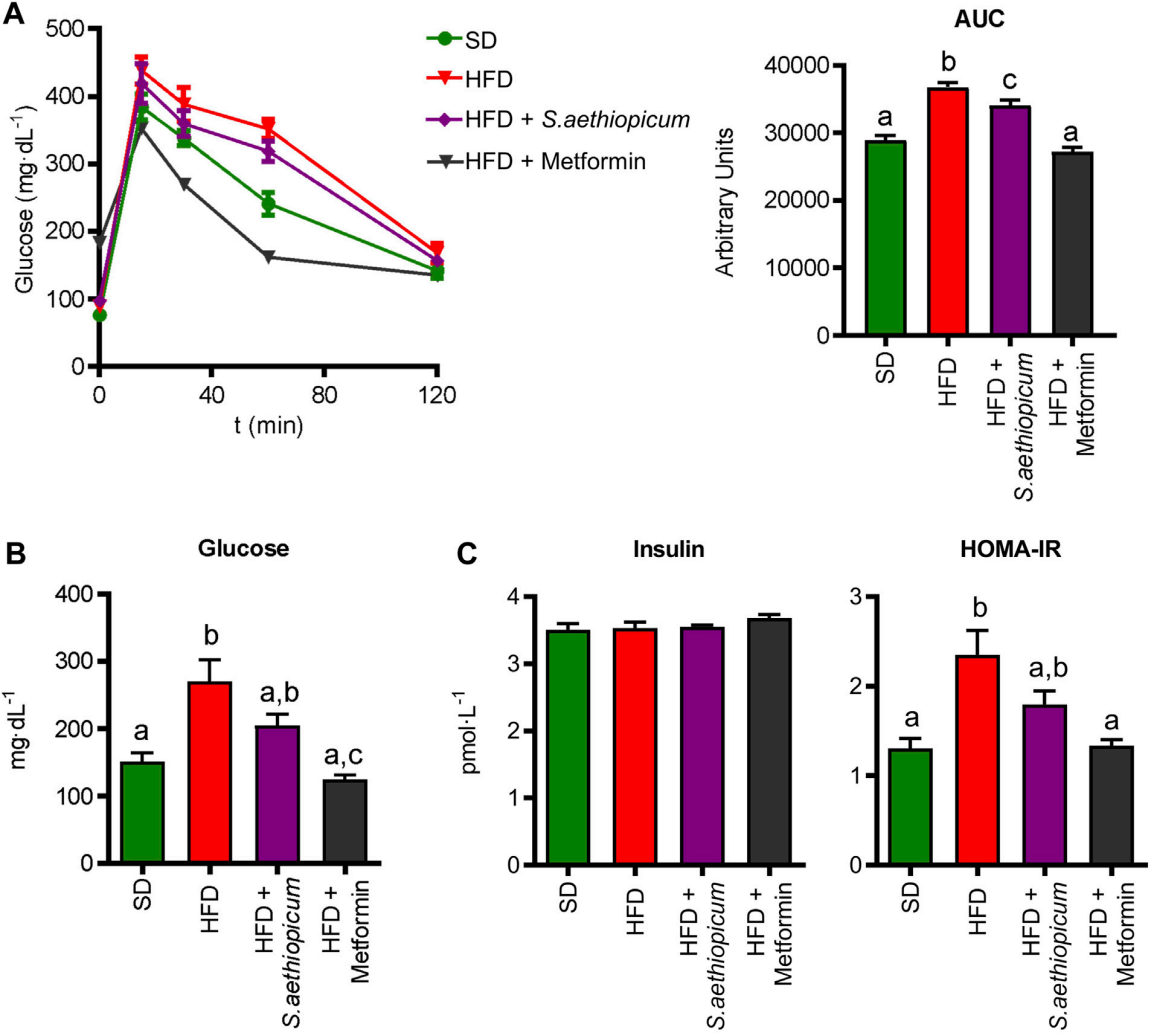
Impact of *S. aethiopicum* peel extract and metformin on **(A)** glucose tolerance test and the area under the curve (AUC) **(B)** glucose and **(C)** insulin levels, and HOMA-IR index in standard (SD) and High-Fat Diet (HFD)-fed mice. Data are expressed as means ± SEM (n = 8). Groups with different letters statistically differ (*p < 0.05*).

Some previous obesity and diabetes preclinical studies in mice and rats have reported the effects of *S. aethiopicum* leaves and fruits extracts to promote a reduction in weight gain (Akinwunmi and Ajibola, 2018; Tuem et al., 2021; Asuquo et al., 2022). This ability of eggplant extracts to reduce weight gain has been mainly attributed to the high presence of polyunsaturated fatty acids together with the ability of different phenolic compounds to inhibit key enzymes involved in lipogenesis and cholesterol synthesis, carbohydrates digestion and glucose absorption (such as α-amylase and α-glucosidase), as previously demonstrated in the *in vitro* assays. Similarly, a decreased activity of *α*-amylase and *α*-glucosidase activities had been also confirmed in rats receiving a fruit African *S. aethiopicum* extract (Nwanna et al., 2016)*.* Additionally, the inhibition of these enzymatic activities has been shown to be associated with reduced glycemic values in diabetes (Tuem et al., 2021). In this regard, *S. aethiopicum* extract positively impacted glucose metabolism, as evidenced in the glucose tolerance test performed 1 week before mice sacrifice (Figure 4A). Concisely, it was able to reduce glycemic levels in obese mice, which resulted in lower values of the area under the curve (AUC) (Figure 4A). As expected, and according to these results, plasma glucose concentrations from HFD control animals were markedly increased compared to SD mice, but Lucanian *S. aethiopicum* extract significantly decreased them (Figure 4B). Of note, although insulin blood levels were the same in the three experimental groups, the administration of *S. aethiopicum* peel extract to HFD-fed mice resulted in a reduction of the HOMA-IR index (Figure 4C), thus indicating an amelioration in obesity-associated insulin resistance. Similarly, metformin administration showed a significant amelioration of the parameters evaluated.

**FIGURE 5:**
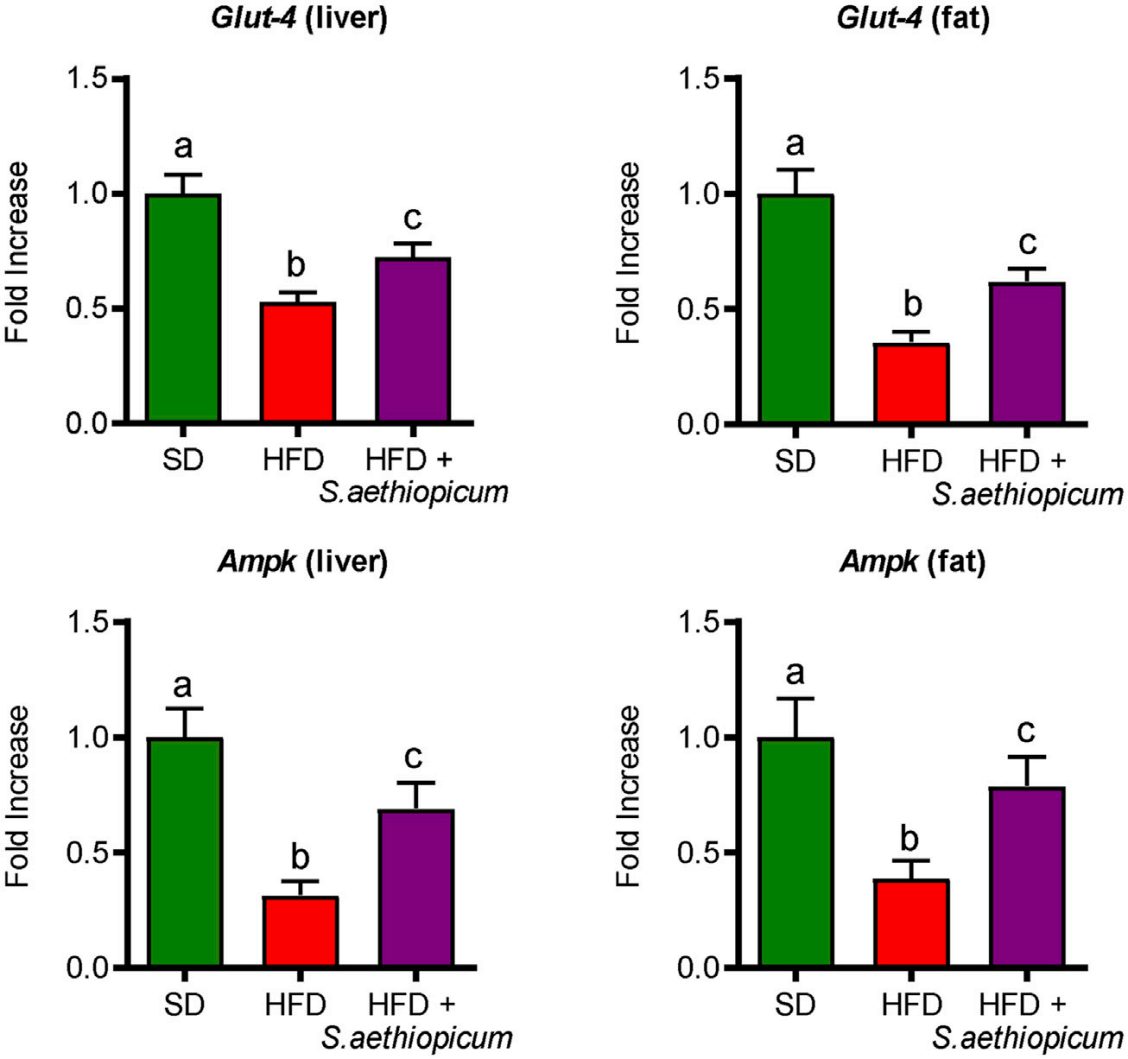
Effects of *S. aethiopicum* peel extract on fat and liver gene expression of *Glut-4* and *AMPK* in standard (SD) and High-Fat Diet (HFD)-fed mice. Data are expressed as means ± SEM (n = 8). Groups with different letters statistically differ (*p < 0.05*).

These results support previous *in vivo* studies reporting that the administration of three different African eggplant fruits (*Solanum kumba*, *Solanum gilo*, and *Solanum aethiopicum*) to diabetic rats significantly reduced blood glucose levels (Nwanna et al., 2016). Similarly, three additional assays revealed that the administration of either leaves or fruits extracts from African *S. aethiopicum* showed hypoglycemic activity in rats (Okafor et al., 2016; Akinwunmi and Ajibola, 2018; Tuem et al., 2021). In consequence, the presence of polyphenolic compounds in the extract, not only downregulates the hyperglycemia in obese mice by inhibiting *α*-amylase and *α*-glucosidase activities but also ameliorates the obesity-associated insulin resistance, as previously proposed for this crude drug (Aryaeian et al., 2017). Furthermore, different phenolic acids like caffeic acid, ellagic acid, or chlorogenic acid, which are present in the Lucanian *S. aethiopicum* peel extract, have been shown to improve glucose metabolism and insulin activity in experimental diabetes, by modulating GLUT expression and promoting its translocation *via* AMP-activated protein kinase (AMPK) and PI3K/Akt pathways (Vinayagam et al., 2016). AMPK is a serine/threonine kinase considered one of the most important indicators of cellular energy status. This enzyme promotes glucose uptake through the translocation of GLUT-4 to the plasmatic membrane and inhibits energy-consuming pathways such as cholesterol synthesis (Habegger et al., 2012); these processes are reduced in obesity and contribute to the development of insulin resistance. In the present study, decreased expression of *Ampk* and *Glut-4* in both hepatic and adipose tissues was observed in control HFD-fed mice, thus explaining the observed impairment in glucose metabolism and insulin sensitivity (Figure 5). When obese mice were treated with the Lucanian *S. aethiopicum* peel extract, there was a significant amelioration in the expression of both *Glut-4* and *Ampk* in tissue levels, being associated with the improved glucose homeostasis and insulin resistance previously discussed (Figure 5).

**FIGURE 6:**
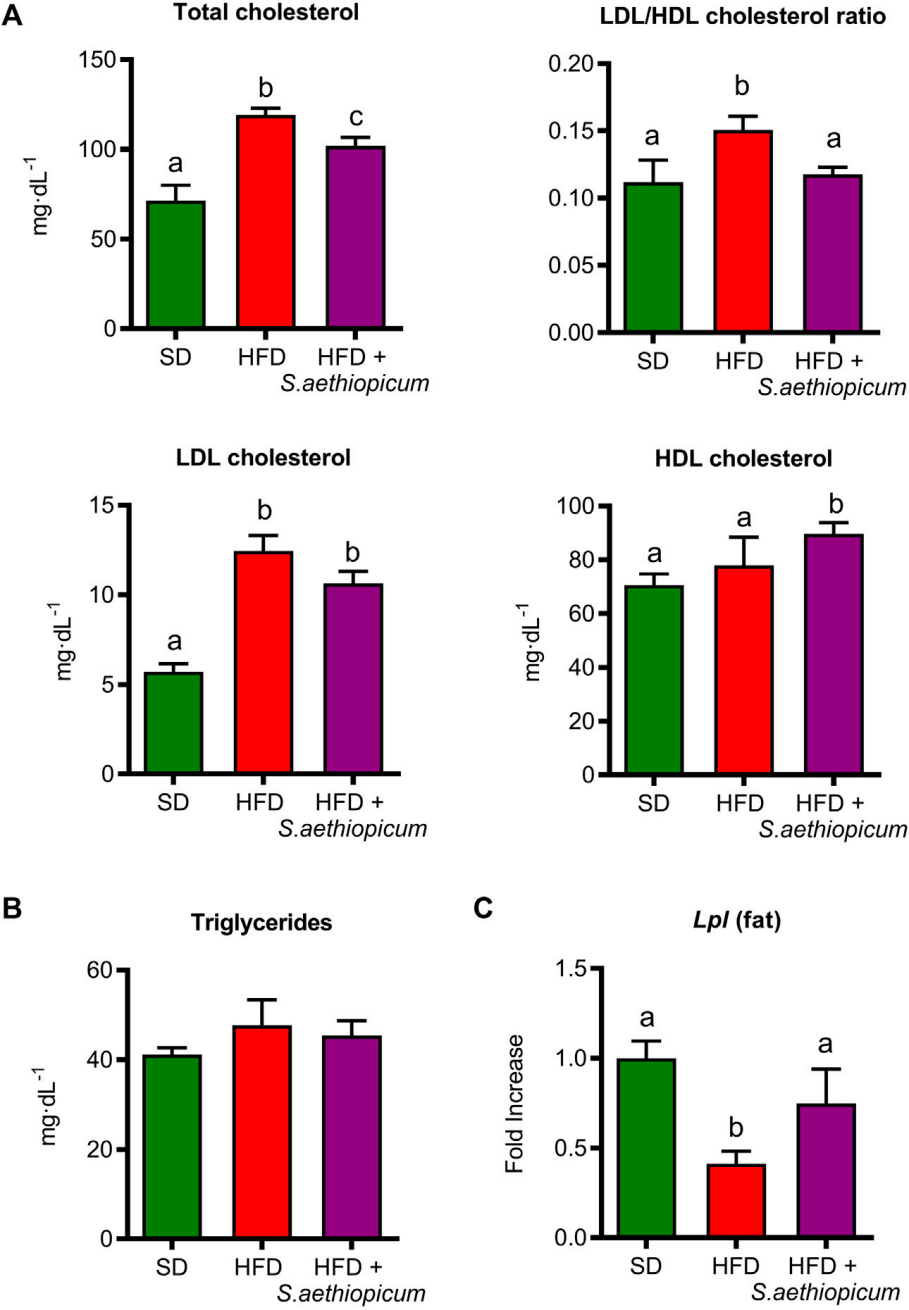
Effects of *S. aethiopicum* peel extract on **(A)** total cholesterol, LDL/HDL ratio, LDL and HDL cholesterol **(B)** triglycerides levels, and **(C)** gene expression of *Lpl* in adipose tissue in standard (SD) and High-Fat Diet (HFD)-fed mice. Data are expressed as means ± SEM (n = 8). Groups with different letters statistically differ (*p < 0.05*).

Insulin resistance may also drive the development of dyslipidemia, typically characterised by increased levels of triglycerides and low-density lipoprotein cholesterol (LDL), as well as by reduced levels of high-density lipoprotein cholesterol (HDL) (Vekic et al., 2019). The present study showed an increase in total cholesterol, LDL/HDL ratio, LDL, and HDL concentration in control HFD-fed mice (Figure 6A). However, the administration of *S. aethiopicum* peel extract did not reduce these parameters, showing no differences in LDL/HDL ratio compared to the SD-fed group (Figure 6A).

The beneficial effects of this eggplant extract on dyslipidemia confirmed previous reports in which the administration of other eggplant extracts obtained from fruits (*S. kumba*, *S. aethiopicum*, and *S. gilo*) to HFD-fed rats resulted in a significant improvement in the lipid profile (Akinwunmi and Ajibola, 2018; Nwanna et al., 2019b). It is well known that lipoprotein metabolism is mainly regulated by the lipoprotein lipase (LPL), an essential enzyme involved in the hydrolysis of triglycerides-rich lipoproteins, being its downregulation linked to a hypertriglyceridemia condition (Noh et al., 2006; Xiao et al., 2017). Specifically, LPL breaks down triglycerides into two free fatty acids and one monoacylglycerol molecule, which can enter the cell and be oxidised by *β*-oxidation (skeletal and cardiac muscle) or resynthesized into triglycerides in adipose tissue and then accumulated. Due to its important function in adipose storage, LPL has also been known as the “gatekeeper of the adipocyte”. Several data reported that body weight gain is inversely associated with LPL mRNA levels, while only a slight reduction was seen in the case of insulin resistance (Kern et al., 1990; Clemente-Postigo et al., 2011). Accordingly, in the present study, although no differences were observed in the concentration of triglycerides (Figure 6B), a significant reduction in *Lpl* expression was shown in HFD-mice while the extract significantly increased the expression of this marker (Figure 6C). Of note, it has been demonstrated that weight loss is linked to increased *Lpl* expression and activity (Kern et al., 1990). The beneficial effects exerted by *S. aethiopicum* peel extract on lipid metabolism in obesity can be associated with the presence of phenolic acids like caffeic acid or gallic acid. In particular, gallic acid regulates AMPK activity by activating the peroxisome proliferator-activated receptor-γ coactivator 1α (PGC1α) (Doan et al., 2015). On the other hand, caffeic acid, through the activation of AMPK, can inhibit the nucleus translocation of Sterol Regulatory Element-Binding Protein-1c (SREBP-1c), leading to lipogenesis and fatty acid synthesis impairment (Ajiboye et al., 2019; Lela et al., 2023). In line with these results, our recent study with the Caco-2 and HepG2 cell lines showed that the Lucanian *S. aethiopicum* peel extract was able to improve lipid absorption and reduce lipid accumulation through the modulation of SREBP-1c and 3-hydroxy-3-methylglutaryl coenzyme A (HMG-CoA) reductase (Lela et al., 2023).

#### 3.3.2 Effects of *S. aethiopicum* peel extract on obesity-related inflammatory markers

**FIGURE 7:**
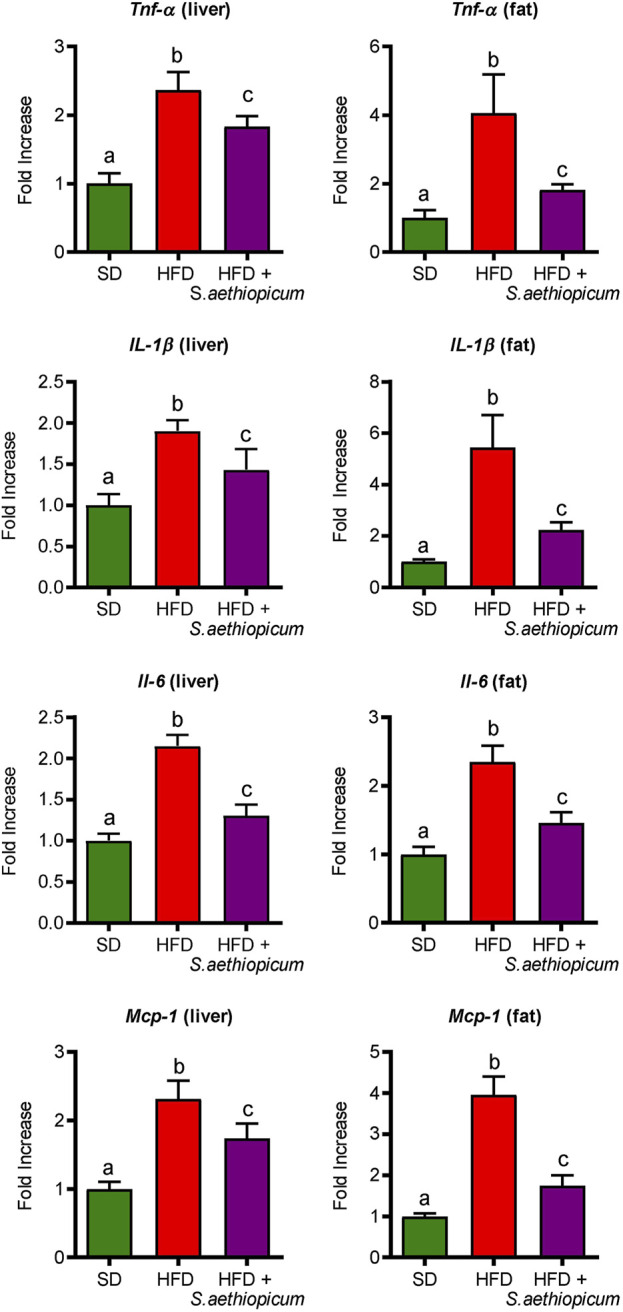
Effects of *S. aethiopicum* peel extract on liver and fat gene expression of *Tnf-α*, *Il-1β*, *Il-6*, and *Mcp-1* in standard (SD) and High-Fat Diet (HFD)-fed mice. Data are expressed as means ± SEM (n = 8). Groups with different letters statistically differ (*p < 0.05*).

**FIGURE 8:**
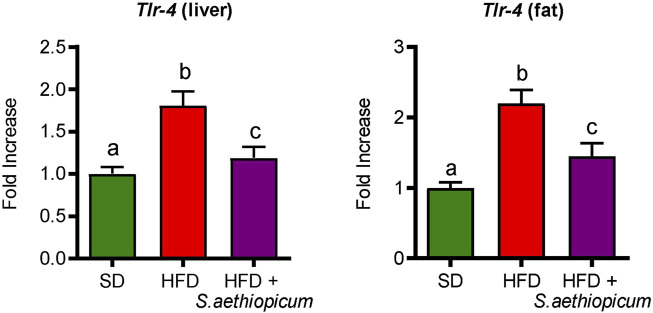
Effects of *S. aethiopicum* peel extract on liver and fat gene expression of *Tlr-4* in standard (SD) and High-Fat Diet (HFD)-fed mice. Data are expressed as means ± SEM (n = 8). Groups with different letters statistically differ (*p < 0.05*).

Obesity is associated with a low-grade inflammation of white adipose tissue (WAT), resulting from chronic activation of the innate immune system (Bastard et al., 2006). Macrophage infiltration into WAT, along with hyperplasia and hypertrophy of adipocytes, results in a local and chronic inflammatory state, evidenced by increased production and secretion of a variety of inflammatory molecules, including tumour necrosis factor-*α* (TNF-*α*), interleukin 6 (IL-6), inflammasome-activated IL-1𝛽, and the monocyte chemotactic protein 1 (MCP-1) among others (Cancello et al., 2005; Rodríguez-Hernández et al., 2013). All these mediators work tightly to establish a vicious circle that activates different inflammatory pathways in both the liver and fat tissues. Accordingly, control HFD-fed mice showed increased mRNA expression of different pro-inflammatory cytokines such as *Tnf-α*, *Il-1β,* and *Il-6*, as well as of the chemokine *Mcp-1* in both hepatic and adipose tissues (Figure 7). With regard to the latter, MCP-1 is normally secreted by adipocytes during obesity-associated inflammation, which typically results in the recruitment of monocytes from the bloodstream, where they differentiate into macrophages (Engin and Engin, 2017). After administration of *S. aethiopicum* peel extract to HFD-mice, the expression of all these inflammatory markers was significantly reduced (Figure 7). The amelioration of the obesity-associated inflammatory state was in accordance with the data discussed above and related to the increased expression of the *Glut-4* receptor (Figure 5). Indeed, it is known that pro-inflammatory cytokines are involved in the development of obesity-related systemic insulin resistance by interfering with insulin receptor function and reducing the expression and activation of glucose transporters such as GLUT-4 (Shepherd and Kahn, 1999). This anti-inflammatory activity exerted by eggplant extracts was also demonstrated in previous *in vitro* and *in vivo* studies performed with the Lucanian *S. aethiopicum* peel extract and the African fruit extract, respectively (Anosike et al., 2012; Nwanna et al., 2019b; Lela et al., 2023), and may be very likely associated with its content in carotenoids, flavonoids and other polyphenols, that among others, target the Toll-like receptor (TLR)-4/NFkB signalling pathway (Lela et al., 2023). In fact, the presence of lycopene and *β*-carotene in *S. aethiopicum* peel extract might contribute to the beneficial effects on the systemic inflammatory response showed in HFD-fed mice by lowering the release of the pro-inflammatory cytokines, as demonstrated in other studies on inflammatory-associated conditions, like LPS-induced septic shock or obesity (Novoselova et al., 2009; Fenni et al., 2017). Similarly, polyphenols have demonstrated their ability to regulate the immune system through the modulation of gene expression and the subsequent synthesis of pro-inflammatory cytokines (Yahfoufi et al., 2018). In addition, TLR signalling also plays a role in the development of obesity-related low-grade inflammation. In fact, TLR signalling regulates the innate immune response in the presence of free fatty acids, which are known to bind either TLR-2 or TLR-4, thus representing a molecular link between hyperlipidemia and the immune system (O'Rourke, 2009). TLR-2 and TLR-4 have also been implicated in glucose metabolism and type 2 diabetes. Mice deficient in these genes were found to be protected against obesity and diet-induced insulin resistance, whereas in humans, TLR-4 gene polymorphisms were associated with a lower risk of type 2 diabetes (Strodthoff et al., 2015). Increased expression of *Tlr-4* in the liver and adipose tissue of obese HFD-fed mice compared to the control group was observed in this study (Figure 8). In agreement with the reduction of the above-mentioned inflammatory parameters and hyperlipidemia, the administration of *S. aethiopicum* peel extract treatment to HFD-fed mice significantly reduced the expression of TLR-4 in both liver and adipose tissue (Figure 8).

**FIGURE 9:**
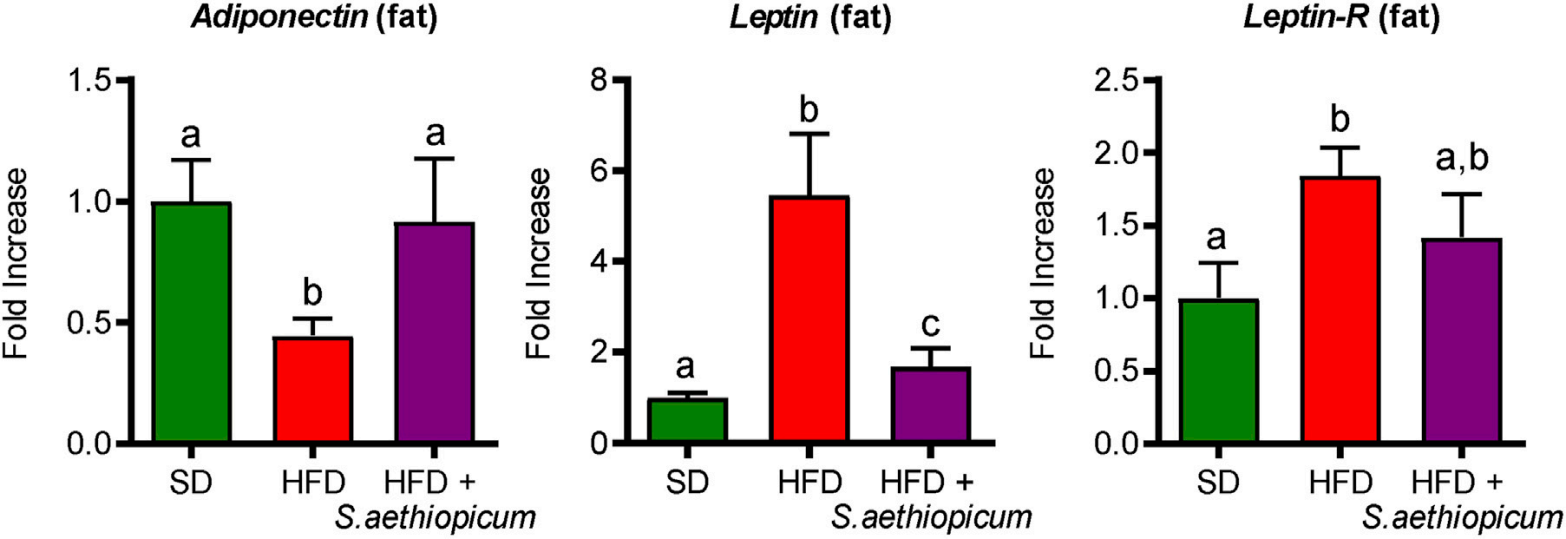
Impact of *S. aethiopicum* peel extract on fat gene expression of *Adiponectin*, *Leptin*, and *Leptin-R* in standard (SD) and High-Fat Diet (HFD)-fed mice. Data are expressed as means ± SEM (n = 8). Groups with different letters statistically differ (*p < 0.05*).

Furthermore, obesity has also been associated with altered production of adipokines by adipose tissue, such as leptin and adiponectin. Leptin shows pro-inflammatory activity because of its capacity to stimulate the proliferation of monocytes and their differentiation into macrophages, modulating the activation of natural killer lymphocytes or inducing the production of pro-inflammatory cytokines such as TNF-α, IL-6, or IL-12. It is well known that leptin levels in adipose tissue and plasma are related to the amount of energy stored in fat tissue depots and the energy balance status, being these levels increased in obese subjects (Sáinz et al., 2015; Obradovic et al., 2021). Instead, adiponectin is an anti-inflammatory adipokine whose bloodstream concentration is strongly influenced by fat mass, since a decrease in obese individuals compared with lean individuals has been observed (Ouchi et al., 2011). As expected, in this study, the expression of both *Leptin* and *Adiponectin* in the adipose tissue was impaired in HFD-fed mice compared to those fed a standard diet (Figure 9). Furthermore, the increased expression of *Leptin* in HFD-fed mice was associated with a reduction in the expression of Leptin receptor (L*eptin-R*) (Figure 9), thus confirming the leptin signalling impairment and the consequently observed hyperleptinemia (Sáinz et al., 2015). However, the administration of *S. aethiopicum* peel extract to HFD-fed mice resulted in a significant improvement in the expression of these adipokines in the fat tissue, (Figure 9). This regulatory activity on adipokine levels could be related to the presence of phenolic acids, like chlorogenic acid, or flavonols, such as quercetin, which are known to reduce leptin levels and increase adiponectin release (Meng et al., 2013; Yahfoufi et al., 2018).

#### 3.3.3 Effects of *S. aethiopicum* peel extract on endothelial dysfunction

**FIGURE 10:**
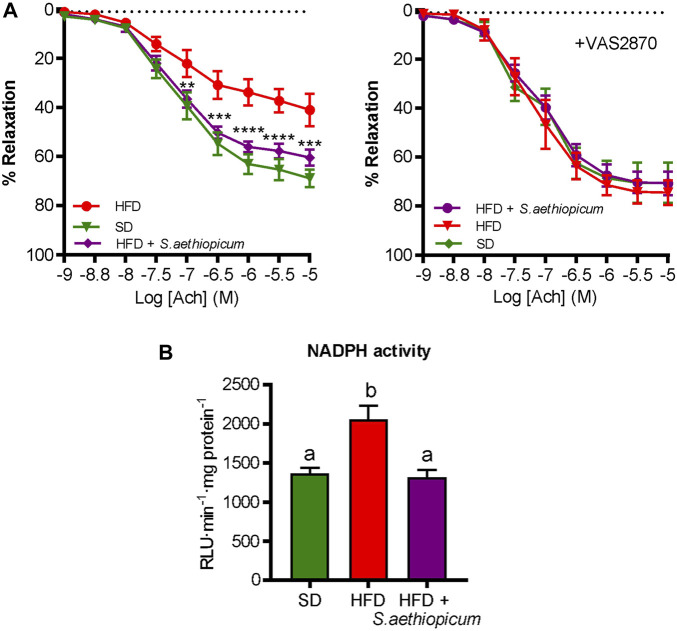
Effects of *S. aethiopicum* peel extract on aortic endothelial function: **(A)** endothelium-dependent relaxation to acetylcholine in the absence or the presence of NADPH oxidase inhibitor VAS2870; **(B)** aortic NADPH activity. Relaxant responses to acetylcholine were expressed as a percentage of pre-contraction induced by U46619. Data are expressed as means ± SEM. Groups with different letters statistically differ (*p < 0.05*); **p < 0.01, ***p < 0.001 and ****p < 0.0001 vs. HFD-fed mice.

Obesity-associated inflammatory state is a leading risk factor for various cardiovascular disorders, which are considerably associated with impaired vascular homeostasis and endothelial dysfunction (Iantorno et al., 2014). Although previous studies have evaluated the cardiac risk ratio by the estimation of atherogenic coefficient and lee index in obese rats treated with an extract of the fruit of *S. aethiopicum* (Akinwunmi and Ajibola, 2018), our study evaluated the functionality of the endothelium, measuring the vasodilator response to acetylcholine of aortic rings, thus representing, as far as we know, the first study to address the effect of *S. aethiopicum* peel extract on endothelial dysfunction. According to previous studies, the HFD-fed group showed a decrease in acetylcholine-induced vasodilatory responses compared to SD-fed mice, thus evidencing an endothelial dysfunction (Figure 10). Interestingly, mice treated with the extract showed an enhancement of endothelial relaxation. When the aortic rings were previously incubated with the NADPH oxidase inhibitor VAS2870, no marked differences in the maximal relaxant response were observed among the experimental groups (Figure 10A).

Also, the activity of the NADPH enzyme, the main source of reactive oxygen species (ROS), was measured to analyse the oxidative stress. It is well reported that ROS are key regulators of vascular health and associated diseases (Chen et al., 2018). Indeed, physiological levels of ROS are responsible for normal smooth muscle cell contraction and endothelial homeostasis. Nevertheless, excessive ROS generation can promote lipid peroxidation, reduced nitric oxide (NO) availability (NO) (a potent vasodilator), the recruitment of inflammatory cells, and the consequent cardiovascular complications characterised by functional and structural vascular cell alterations. As expected, obese mice exhibited a more pronounced NADPH activation than the lean mice, while the treatment with the extract was able to downregulate it (Figure 10B). This suggested a restoration of NO bioavailability and a reduction of ROS production, which could justify the improvement of the impaired endothelium-dependent relaxation to acetylcholine.

## 4 Concluding remarks

*S. aethiopicum* peel extract from the Basilicata Region (Italy) contains a rich source of active molecules such as phenolic acids (caffeic acid, gallic acid, ellagic acid, chlorogenic acid), flavonols (quercetin, rutin, kaempferol), flavanols (epicatechin), anthocyanins (delphinidin-3-rutinoside), vitamins (ascorbic acid), carotenoids (*β*-carotene, lycopene), and sesquiterpenoids (solavetivone). Furthermore, the extract showed its ability to inhibit α-glucosidase and α-amylase enzymatic activities. Additionally, this *S. aethiopicum* peel extract ameliorated HFD-induced obesity in mice, by reducing weight gain and the associated hyperplasia of adipose tissue and improved both glucose and lipid metabolism. These beneficial effects were associated with an improvement in the chronic low-grade inflammation and vascular dysfunction, typically associated with this condition. Based on these promising results, the Lucanian *S. aethiopicum* peel extract could represent a source of natural compounds for the treatment of obesity, hypertension, and cardiovascular conditions.

## Data Availability

The original contributions presented in the study are included in the article/Supplementary Materials, further inquiries can be directed to the corresponding authors.
