# Supplementary material for: Solanum aethiopicum L. from the Basilicata region as a source of specialized metabolites with promising anti-obesity effects: phytochemical characterization and in vivo investigation in high fat diet-fed mice
Source: Front Pharmacol. 2023 Nov 22;14:1306135. doi: 10.3389/fphar.2023.1306135 (PMC10702577; doi:10.3389/fphar.2023.1306135)
Supplement: Supplementary file 1 [file DataSheet1.PDF]

Expanded Materials and Methods

Glucose Tolerance Test, Plasma determinations and Morphological Variables

A glucose tolerance test was carried out one week before the end of the experiment. Mice fasted for 18 h received a glucose solution diluted in water (2 g/kg) by intraperitoneal injection, and blood was collected from the tail vein at 0, 15, 30, 60 and 120 min after injection. At the end of the treatment, mice were sacrificed under isoflurane anaesthesia. Subsequently, plasma blood samples were collected and frozen at −80 °C to measure glucose, total cholesterol, high-density lipoprotein (HDL) cholesterol, low-density lipoprotein (LDL) cholesterol, and triglycerides by using colorimetric methods (Spinreact kits) (Spinreact, S.A., Girona, Spain). Plasma insulin concentrations were quantified using a mouse insulin ELISA kit (Alpco Diagnosis, Salem, NH, USA). Homeostasis model assessment of insulin resistance (HOMA-IR) was calculated using the formula: fasting glucose (mM)×fasting insulin (μ-units/mL)/22.5. Hepatic and adipose tissue were extracted to evaluate gene expression by RT-qPCR. Briefly, total RNA from liver and fat samples was extracted using TRIzol® Reagent (Invitrogen Life Technologies, Carlsbad, CA, USA) following manufacturer's instructions. RNA was transcribed using oligo(dT) primers (Promega, Southampton, UK), and the resulting cDNA (20 ng) was amplified on optical grade 48-well plates in an Eco™ Real-time PCR system (Illumina, San Diego, CA, USA) using KAPA SYBR® FAST qPCR Master Mix (Kapa Biosystems, Wilmington, MA, USA). The specific primers used for each gene are shown in Table S1. To normalise mRNA expression, the expression of the housekeeping gene glyceraldehyde 3-phosphate dehydrogenase (*Gapdh*) was measured for comparative reference. The relative gene expression was calculated using the ΔΔCt method.

Vascular reactivity studies and NADPH oxidase activity

The obesity-associated vascular dysfunction was performed in descending thoracic aortic rings by measuring acetylcholine vaso-relaxant ability and NADPH oxidase activity.
For the vascular reactivity study, the organ chamber was filled with Krebs solution (composition in mM: KH_2PO 1.2$_4$, $CaCl_2$ 2, 1, $MgSO_4$ 1.2, $NaHCO_3$ 25, KCl 4.75, NaCl 118, and glucose 11) at 37 °C and gassed with 5 % CO_2 and 95 % O_2 (pH 7.4). Length-tension properties were assayed via the myograph software (Myodaq 2.01 (Danish Myo-technologies, Denmark)) and the aortae were loaded to a tension of 5 mN. After stabilisation, cumulative concentration-response curves to acetylcholine (10^{-9} M-10^{-5} M) were performed in intact rings pre-contracted by U46619 (10^{-8} M) in the absence or in the presence of the selective nicotinamide adenine dinucleotide phosphate (NADPH) oxidase inhibitor VAS2870 (10^{-5} M), which was added 30 min before. Relaxant responses to acetylcholine were expressed as a percentage of pre-contraction induced by U46619. The evaluation of NADPH oxidase activity in aortic rings was determined by lucigenin-enhanced chemiluminescence assay. Aortic rings were incubated for 30 min at 37 °C in HEPES-containing physiological salt solution (pH 7.4; in mM: $CaCl_2$ 1.2, $NaHCO_3$ 1, KH_2PO_4 0.4, Na_2HPO_4 0.15, KCl 4.6, HEPES 20, NaCl 119, $MgSO_4$ 1, and glucose 5.5). To stimulate the aortic production of O^{2-}, the rings were incubated with NADPH (100 μM). Consequently, the aortic rings were then placed in tubes containing physiological salt solution, with or without NADPH and lucigenin was injected automatically at a final concentration of 5 μmol/L. NADPH oxidase activity was obtained by measuring luminescence over 200 s in a scintillation counter (Lumat LB 9507, Berthold, Germany) in 5-s intervals and was calculated by subtracting the basal values from those in the presence of NADPH. Vessels were then dried, and dry weight was determined. NADPH oxidase activity is defined as relative luminescence units (RLU)/min/mg dry aortic ring.

Table S1. RT-qPCR primers sequences.

Gene	Organism	Sequence 5'- 3'	Annealing T °C	Accession number
Gapdh	Mouse	FW: CCATCACCATCTTCCAGGAG<br>RV: CCTGCTTCACCACCTTCTTG	60	NM_001289726.1
Adipoq	Mouse	FW: GATGGCAGAGATGGCACTCC<br>RV: CTTGCCAGTGCTGCCGTCAT	52	NC_000082.7
Ampk	Mouse	FW: GACTTCCTTCACAGCCTCATC<br>RV: CGCGCGACTATCAAAGACATACG	60	XM_036159053.1
Glut4	Mouse	FW: GAGAATACAGCTAGGACCAGTG<br>RV: TCTTATTGCAGCAGCGCCTGAG	62	NC_000077.7
Il1β	Mouse	FW: TGATGAGAATGACCTCTTCT<br>RV: CTTCTTCAAAGATGAAGGAAA	60	NC_000068.8
Il6	Mouse	FW: TAGTCCTTCCTACCCCAATTTCC<br>RV: TTGGTCCTTAGCCACTCCTTCC	60	NM_031168.2
Lep	Mouse	FW: TTCACACACGCAGTCGGTAT<br>RV: GCTGGTGAGGACCTGTTGAT	60	NC_000072.7
Lepr	Mouse	FW: GCTATTTTGGGAAGATGT<br>RV: TGCCTGGGCCTCTATCTC	60	NC_000070.7
Lpl	Mouse	FW: TTCCAGCCAGGATGCAACA<br>RV: GGTCCACGTCTCCGAGTCC	60	NC_000074.7
Mcp1	Mouse	FW: AGCCAACTCTCACTGAAG<br>RV: TCTCCAGCCTACTCATTG	55	NC_000077.7
Tlr4	Mouse	FW: GCCTTTCAGGGAATTAAGCTCC<br>RV: AGATCAACCGATGGACGTGTAA	60	NM_021297.3
Tnfα	Mouse	FW: AACTAGTGGTGCCAGCCGAT<br>RV: CTTCACAGAGCAATGACTCC	60	NM_001278601.1
